# Postoperative Seizure Rate After Transcortical Resection of Subcortical Brain Tumors and Colloid Cysts: A Single Surgeon’s Experience

**DOI:** 10.7759/cureus.2115

**Published:** 2018-01-26

**Authors:** Daniel G Eichberg, Shaina Sedighim, Simon Buttrick, Ricardo J Komotar

**Affiliations:** 1 Neurological Surgery, Univeresity of Miami Miller School of Medicine; 2 Neurological Surgery, University of Miami Miller School of Medicine

**Keywords:** transcortical approach, transcallosal approach, transsulcal approach, brain tumor, colloid cyst, neurosurgery, seizure

## Abstract

When deciding on a surgical route to reach subcortical brain tumors and colloid cysts, many surgeons advocate the use of transcallosal, transsulcal, or skull base approaches over transcortical approaches due to a high reported incidence of postoperative seizures. We have retrospectively analyzed all patients operated upon by a senior neurosurgeon (Ricardo J. Komotar) who undertook transcortical approaches for the resection of subcortical brain tumors and colloid cysts. We have also performed a comprehensive review of the literature to estimate postoperative seizure risk after transcortical approaches for the resection of deep tumors and colloid cysts. Of 27 patients who underwent transcortical approaches for the resection of subcortical brain tumors and colloid cysts, zero had postoperative seizures. A comprehensive review of the literature shows an 8.3% postoperative risk of seizures after the transcortical approach. Our institution has never experienced a postoperative seizure following the transcortical approach for the resection of deep tumors and colloid cysts. For this reason, we advocate selecting a surgical approach that obtains adequate lesion exposure and minimizes the violation and retraction of eloquent cortex, venous structures, and white matter tracts, rather than on presumed postoperative seizure risk.

## Introduction

The resection of deep-seated intraventricular and subcortical brain tumors is a difficult task, as complete resection requires the surgeon to develop a corridor that will minimize eloquent brain compromise and the retraction of parenchyma. The transcortical approach for the removal of such tumors was initially preferred by surgeons because it offers a wide microsurgical working space and flexibility, as seen in the resection of lateral ventricular tumors [1]. Despite the efficacy and ease of the transcortical approach, many have argued against its use due to a perceived risk of postoperative epilepsy [2].

In order to avoid post-operative complications, many surgeons favor the transcallosal and skull-based approaches over the transcortical approaches due to a reportedly lower seizure rate. Transcallosal approaches have been shown to decrease the risk of seizures and functional deficits in a variety of tumor types, including lateral ventricular tumors and colloid cysts of the third ventricle [3-4]. For third ventricular colloid cyst resections, the greatest advantage of the transcallosal approach is the avoidance of a cortical incision and the ability to provide natural planes for dissection to the anterior portion of the third ventricle. Nevertheless, there are technical hurdles surgeons must overcome when implementing this approach, including the varying anatomy of the cortical bridging veins, the dissection of the pericallosal arteries, and the need for callosal sectioning [5]. Recently, there has been much interest in endoscopic approaches for the removal of colloid cysts, as this is a less invasive technique and may minimize post-operative complications and preoperative morbidity and allow for shortened hospital stays [6-8]. However, endoscopic approaches have their drawbacks, as they require a steep learning curve to master and may be associated with greater residual lesion and recurrence rates [9-10].

Here, we present our institution’s experience with transcortical approaches for subcortical lesions, as well as a review of the pertinent literature examining the seizure and complication rates of transcortical approaches.

## Materials and methods

Patient selection

All patients undergoing transcortical resection of a subcortical lesion (defined as any lesion greater than one centimeter below the cortical surface) at the University of Miami Hospital from June 2015 to February 2017 were included. The senior neurosurgeon’s (Ricardo J. Komotar) clinical database was searched to obtain all relevant patients. This study was approved by the institutional review board, along with a waiver of informed consent.

Surgical technique

In the majority of cases, we use a tubular retractor technique to access deep-seated lesions via a transcortical approach in order to minimize retractor-related rain injury, as tubular retractors distribute pressure radially [11]. During preoperative trajectory planning, surface anatomic landmarks are used to identify and avoid eloquent brain areas. If necessary, diffusion tensor imaging (DTI) is used for white matter tractography to minimize damage to eloquent white matter pathways. The entry point is chosen with neuronavigation assistance, and an approximately 3 cm craniotomy is turned centered around this point. An extensive sulcal dissection is performed centered on the entry point whenever possible to lessen the amount of healthy brain parenchyma violated during tubular retractor advancement. If the planned trajectory is prohibited by intervening venous structures, a small corticotomy is made, and the tubular retractor is inserted. Then, the standard bimanual microsurgical technique is utilized to resect the lesion through the tubular retractor system. For less dense lesions, dissection is initiated at the distal deep component of the lesion and continues proximally toward the surgeon. In contrast, for firmer lesions, the retractor is placed on the proximate tumor edge and dissection and tumor resection continues distally. The tubular retractor may be safely angled if needed to enlarge the field of view for large tumors. To confirm gross total or maximal safe resection, an angled endoscope is used to circumferentially inspect the resection bed, thus overcoming the field-of-view restriction imposed by the retractor. Finally, hemostasis is achieved along the surgical corridor, as the tube is the tube is progressively removed.

Postoperative medical management

Postoperative, patients are placed on levetiracetam for seizure prophylaxis for two weeks. For treatment of cerebral edema, patients with supratentorial lesions are placed on dexamethasone with a one-week taper and patients with eloquent area lesions or infratentorial lesions with a two-week dexamethasone taper. Patients with benign lesions have dexamethasone tapered to off, and those with malignant lesions have dexamethasone tapered to 2 mg BID.

Seizure evaluation

Postoperative seizures were defined as clinically witnessed seizure events as determined by board-certified neurosurgeons. If patients were to develop clinical seizures, they would be monitored by electroencephalogram placement.

Clinical data acquisition

The electronic medical record was analyzed to determine patient demographic data, operative information, and post-operative course.

Literature analysis

A comprehensive review of the literature was performed using the key words “transcortical,” “deep-seated brain lesion,” “transgyral,” “superior frontal gyrus approach,” “middle temporal gyrus approach,” “inferior temporal gyrus,” and “superior parietal lobule approach,” alone or together to search the PubMed, Ovid Medline, Ovid EMBASE, Scopus, and Web of Science databases and all neurosurgical peer-reviewed journals. Inclusion criteria included the following: English language, use of transcortical approach, discussion of seizure risk, and deep-seated cranial neoplastic and cystic lesions. Exclusion criteria included exclusive use of transcallosal, endoscopic, or skull base approaches.

## Results

A total of 27 patients underwent transcortical resections of brain tumors, including 13 males and 14 females. The average age was 55 years and the average follow-up was 14.3 months, ranging from 0.25-30 months. The central nervous system tumor pathology was as follows: six patients (22%) presented with colloid cysts; six (22%) had either glioblastoma or grade three gliomas; one patient (3.7%) presented with a low-grade glioma, one patient with a subependymoma, and one patient (3.7%) had a central neurocytoma. Eleven patients (40.7%) had metastases, and, finally, one patient (3.7%) presented with radiation necrosis. Gross total resection was possible in 22 (81.5%) of patients, while the remaining five patients (18.5%) underwent subtotal resection.

In 20 cases, a tubular retractor system was used. Three postoperative complications occurred, including one case of transient short-term memory difficulty, one case of mild speech and short-term memory impairment, and one postoperative stroke of unclear etiology on postoperative day one. No postoperative seizures occurred.

For the literature review, nine papers analyzing 261 patients were found that reported complication rates of transcortical approaches for tumors and colloid cysts (Table 1). Of the 238 cases in which seizure rates were reported, 20 patients (8.3%) had at least one postoperative seizure.

**Table 1:** Literature review of transcortical resection complication rates Abbreviations: DVT: deep venous thrombosis; IPH: intraparynchymal hemorrhage; IVH: intraventricular hemorrhage; NS: not specified

Author	Year	Number of Patients	Approach used, (n).	Percent total gross resection	Seizure rate (%)	Rate of other complications (%)
Quinones-Hinojosa et al. [12]	2017	23	Inferior temporal gyrus, 14; Middle temporal gyrus, 9.	92%	NS	Clinically significant stroke (8.7%); Visual deficits (8.7%); Speech deficits (8.7%).
Sabanci et al. [13]	2017	41	Transcortical	95%	7.3%	Bacterial meningitis (14.6%); Hemiparesis (7.3%).
Asgari et al. [14]	2003	27	Frontal transcortical route via a typical incision over the frontomedian gyrus, 27.	92.6%	26%	Diencephalic injury (22%); Transient mutism (11%); Hemiparesis (7%); Subdural hygroma (30%).
Mazher et al. [15]	2013	33	Transcortical	NS	3%	Persistent postoperative hydrocephalus (27.3%); Ventriculitis (9%); Postoperature neurological deficits (6%); Intraventricular hemorrhage (3%); Subdural hygroma (3%).
Milligan et al. [16]	2009	52	Superior frontal gyrus, transventricular, 28; Superior frontal gyrus, transcapsular, 2; Superior parietal lobule, 17; Middle temporal gyrus, 5.	88%	8%	Aphasia (31%); Altered consciousness (6%); Cognitive/personality (12%); Edema (4%); DVT (4%); Endocrinopathy (4%); Gerstmann syndrome (2%); Hydrocephalus, temporary (10%); Hemiparesis (35%); IPH (2%); Meningitis (6%); IVH (4%); Memory (12%); Neglect (4%); Stroke (1%); Visual field deficit (15%); Ventriculoperitoneal shunt (12%).
Solaroglu et al. [17]	2004	26	Transcortical-transventricular approach through the middle frontal gyrus of the nondominant lobe, 26.	NS	8%	Wound infection (4%).
Ben Nsir et al. [18]	2016	3	Posterior parietal, 2; Right frontal, 1.	100%	0%	Mild hemiparesis (33%); Mild brachial paresis 33%).
D’Angelo et al. [19]	2005	44	Frontal transcortical approach, 12; Middle temporal gyrus approach, 14. Parietal transcortical approach, 7. NS, 11.	NS	5.9%	Intracerebral hematoma (11.4%); Subdural hygroma (4.5%).
Park et al.[20]	2012	12	Frontal Transcortical approach, 12.	100%	0%	Transient hemiparesis (16.7%); Transient aphasia (8.3%); Subdural hygroma (8.3%).

## Discussion

Techniques for the resection of deep-seated tumors and colloid cysts have been debated since the early days of neurosurgery, and the optimal surgical approach continues to be debated. Transcortical approaches for the removal of tumors are often avoided due to reportedly high postoperative seizure rates, theoretically due to cortical retraction, corticotomy, or ischemia and infarction after venous sinus and cortical vein violation [12]. Desai et al. (2002) reported that of the 30 patients operated on by the transcortical-transventricular approach, 26.6% experienced seizures postoperatively [2]. Sabanci et al. (2017) found that although the rates of residual cysts are higher when utilizing endoscopic approaches (versus conventional transcortical approaches), conventional approaches result in a significantly higher (p=0.012) incidence of postoperative seizures when compared to both the mini-tubular and endoscopic methods [13].

Ellenbogen et al. (2001) outlined the neuropsychological, functional, and neurological outcomes of 29 consecutive lateral ventricular tumors resected via the transcortical route over a five-year period. The majority of patients (86%) had acceptable outcomes, returning to baseline functionality and suffering from minimal morbidity; yet, 7% of patients experienced postoperative epilepsy [1]. Similarly, Milligan and Meyer reported an 8% incidence of new seizures with transcortical approaches. Interestingly, they find an even higher rate of postoperative seizures with the transcallosal approach (25%, p<0.05) [12]. 

Compared to the transcortical approach, the transcallosal approach may pose higher rates of venous infarctions during the resection of colloid cysts. Sheikh et al. (2014) suggested that this phenomenon is most likely due to the increased risk of injury to bridging veins when accessing the interhemispheric corridor during transcallosal surgery [8]. When resecting tumors of the lateral ventricles, lesions in the temporal horn or atrium are more safely approached through cortical incisions [1]. Furthermore, the transcortical approach for the resection of ventricular lesions is associated with fewer memory deficits than the transcallosal route, likely due to decreased damage to the fornices [12].

Despite an estimated 8.3% postoperative seizure rate based on a comprehensive review of the literature, none of the 27 patients from our series, who underwent a transcortical resection of a subcortical lesion, experienced a postoperative seizure. Possible reasons for these results include obtaining adequate preoperative seizure control, modern neuroanesthesia techniques, and postoperative seizure prophylaxis in all patients with levetiracetam. Additionally, every effort is made to minimize the size of the corticectomy, especially when using tubular retractors.

A total of 20 cases (74.1%) from our series utilized tubular retractor systems, which may have contributed to our low seizure rate. In contrast to the retractor blades used in open craniotomies, tubular retractors distribute pressure equally and circumferentially, which may minimize the compromise of tissue perfusion and thus reduce local brain tissue injury, infarction, and contusion [11], factors hypothesized to contribute to the reportedly high postoperative seizure rate of the transcortical approach. Additionally, tubular retractors can theoretically allow for the gentle spreading of white matter fibers, thus decreasing the number of tracts that are transected and can usually permit resection through a smaller corticectomy, another factor purported to increase seizure risk [12].

Our study has several important limitations that restrict its generalizability. First, the study is a small retrospective case series with a relatively small sample size of 27 patients. It is conceivable that the study’s low patient population failed to observe a representative number of patients that developed postoperative seizures following the transcortical approach resection of deep tumors, leading to an artificially low postoperative seizure rate. Therefore, larger studies are required to validate our results. Additionally, it is possible that our patient cohort’s lesion pathology profile is skewed toward lesions that are less epileptogenic, thereby artificially decreasing the postoperative seizure rate. Despite these two limitations that may have artificially lowered our reported postoperative seizure rate, the fact that no postoperative seizures were reported suggests that it is at least worthwhile to consider the transcortical approach for the resection of subcortical lesions if it were to create a favorable surgical trajectory. Further, future corroborating, randomized controlled studies would provide more confidence in our results, as randomization would remove a potential source of study bias and a control group would provide a more accurate outcome comparison than previously published data with possibly dissimilar patient populations. Finally, postoperative seizures were defined as clinically witnessed seizure events, rather than an electroencephalographic seizure, as patients were not routinely monitored on electroencephalography (EEG). Although it is possible that some postoperative seizures were missed due to the lack of EEG monitoring, we do not believe this was a significant study limitation, as patients were closely monitored postoperatively in a dedicated neurosurgery intensive care unit by specialized nurses, neurointensivists, and board-certified neurosurgeons, who would likely have detected a clinical seizure.

Because of the low incidence of postoperative seizures complicating transcortical approach surgeries in our experience, we select the surgical approach for subcortical tumors and colloid cysts based on the trajectory that will provide maximal lesion exposure as well as minimal violation of the eloquent cortex, white matter tracts, and venous structures. We do not avoid the transcortical approach solely due to the high reported postoperative seizure risk.

## Conclusions

While a comprehensive review of the literature suggests an 8.3% risk of postoperative seizures following the transcortical approach for the resection of subcortical tumors and colloid cysts, this single surgeon series of 27 consecutive cases does not report any postoperative seizures. For this reason, we advocate determining the surgical approach for a given lesion based on gaining optimal lesion exposure and minimizing damage to the eloquent cortex, venous structures, and white matter tracts, rather than on a presumed postoperative seizure risk.
